# Assessment of the neuroprotective potential of d-cycloserine and l-serine in aluminum chloride-induced experimental models of Alzheimer’s disease: *In vivo* and *in vitro* studies

**DOI:** 10.3389/fnut.2022.981889

**Published:** 2022-09-08

**Authors:** Özlem Özdemir Tozlu, Hasan Türkez, Ufuk Okkay, Onur Ceylan, Cemil Bayram, Ahmet Hacımüftüoğlu, Adil Mardinoğlu

**Affiliations:** ^1^Department of Molecular Biology and Genetics, Erzurum Technical University, Erzurum, Turkey; ^2^Department of Medical Biology, Faculty of Medicine, Atatürk University, Erzurum, Turkey; ^3^Department of Medical Pharmacology, Faculty of Medicine, Atatürk University, Erzurum, Turkey; ^4^Department of Medical Pathology, Faculty of Medicine, Atatürk University, Erzurum, Turkey; ^5^Science for Life Laboratory, KTH-Royal Institute of Technology, Stockholm, Sweden; ^6^Centre for Host-Microbiome Interactions, Faculty of Dentistry, Oral & Craniofacial Sciences, King’s College London, London, United Kingdom

**Keywords:** Alzheimer’s disease, aluminum, D-cycloserine, L-serine, neuroprotective, neuro-nutrient

## Abstract

Alzheimer’s disease (AD) is a neurodegenerative disease characterized by the accumulation of amyloid-β (Aβ) plaques and neurofibrillary tangles in the brain accompanied by synaptic dysfunction and neurodegeneration. No effective treatment has been found to slow the progression of the disease. Therapeutic studies using experimental animal models have therefore become very important. Therefore, this study aimed to investigate the possible neuroprotective effect of D-cycloserine and L-serine against aluminum chloride (AlCl_3_)-induced AD in rats. Administration of AlCl_3_ for 28 days caused oxidative stress and neurodegeneration compared to the control group. In addition, we found that aluminum decreases α-secretase activity while increasing β-secretase and γ-secretase activities by molecular genetic analysis. D-cycloserine and L-serine application resulted in an improvement in neurodegeneration and oxidative damage caused by aluminum toxicity. It is believed that the results of this study will contribute to the synthesis of new compounds with improved potential against AlCl_3_-induced neurodegeneration, cognitive impairment, and drug development research.

## Introduction

Alzheimer’s disease is marked by a gradual loss of neuronal and synaptic functioning, resulting in memory and cognition problems. The major histological hallmarks of Alzheimer’s disease are the deposition of amyloid beta peptides (Aβ) in neuronal cells and the creation of intracellular neurofibrillary tangles (1). The etiology of Alzheimer’s disease is complex; the main pathogenic processes in the disease include oxidative stress, amyloidogenesis, and neuroinflammation (2).

Aluminum (Al), an environmental contaminant, has been implicated in the development of Alzheimer’s disease (3, 4). Al has a neurotoxic-like effect on neuronal structure (5, 6), blood-brain barrier (BBB) permeability, and cholinergic/noradrenergic neurotransmission (7–9). Several investigations have shown that exposure to solid aluminum chloride and its decomposed form (ion metal Al^3+^) can change the BBB, influence axonal transport, and cause inflammatory responses as well as synaptic structural abnormalities, resulting in significant memory loss (4, 7–9). Furthermore, the metal ion Al^3+^ hastens the dynamic process of Aβ aggregation, hence increasing neurotoxicity in neuronal cells as a result of significant changes in the biophysical characteristics of the Aβ peptide, which leads to its accumulation in the cortex and hippocampus (4, 9, 10).

Furthermore, Al causes cytoskeletal proteins to misfold, resulting in the production of amyloid plaques and neurofibrillary tangles in the brain (11, 12). As a result, using Al to induce neurodegenerative changes in animals to mimic Alzheimer’s disease is generally recognized.

A disruption in glutamatergic neurotransmission *via* the N-methyl-D-aspartate (NMDA) subtype of glutamate receptors may be implicated in the etiology of Alzheimer’s disease, according to many lines of evidence (13–15). NMDA receptors are diminished selectively and variably in parts of the brain associated with Alzheimer’s disease (16, 17), suggesting that Alzheimer’s disease may be linked to the loss of NMDA receptors in specific brain regions. Treatment with memantine, an NMDA receptor antagonist, was recently found to minimize clinical deterioration in moderate-to-severe Alzheimer’s disease patients, implying a role for NMDA receptors in the pathogenesis of the disease (18). D-serine has been found to act as an endogenous ligand for the NMDA receptor’s strychnine-insensitive glycine sites (19). Furthermore, free D-serine levels in the frontal cortex of Alzheimer’s patients were comparable to those in the normal brain (20). D-cycloserine, a partial agonist of the NMDA receptor glycine site, has improved memory-related activities in Alzheimer’s patients (21, 22). L-serine is a precursor of D-serine, the synaptic NMDAR’s major coagonist, which is necessary for synaptic activity and plasticity. Therefore, it would be of great interest to clarify the potential contribution of L-serine and D-cycloserine to the pathophysiology of Alzheimer’s disease. Along these lines, the current work focuses on the neuroprotective effects of L-serine and D-cycloserine against AlCl_3_-induced Alzheimer’s disease *in vitro* and *in vivo*. It investigates the effects of L-serine and D-cycloserine on cognitive decline and oxidative stress in animals, as well as histopathological examinations. This research aids in slowing disease development and identifies viable therapeutic targets for treating Alzheimer’s disease.

## Materials and methods

### Cell cultures and cellular differentiation

SH-SY5Y cells of human neuroblastoma origin were cultured in Dulbecco’s modified Eagle medium F12 (Gibco^®^, New York, United States) supplemented with 10% fetal bovine serum (Gibco^®^, New York, United States), 1% penicillin and streptomycin at 37°C in 5% CO_2_. Cells were seeded onto plates and passaged when they reached 70–80% confluence. For the differentiation of SH-SY5Y cells, the medium was replaced with DMEM: F12 medium containing 1% FBS and 10 μM retinoic acid (RA, Sigma–Aldrich^®^, Milan, Italy). The media of the cells were renewed every 3 days with a medium containing 1% FBS and 10 μM RA. The differentiation process of the cells was observed for 11 days with light microscopy (23).

### *In vitro* treatments

#### WST-8 assay

Cell viability was measured by using a CVDK-8 (Ecotech Biotechnology^®^) kit according to the manufacturer’s manual. Briefly, 1 × 10^4^-1 × 10^5^ cells were seeded in 96-well plates and kept under appropriate culture conditions (37°C, 5% CO_2_) for 24 h for cell attachment. Then, the cells were incubated with different concentrations (0-800 μg/ml) of D-cycloserine (DCS) or L-serine (LS, Sigma–Aldrich, St. Louis, MO, United States) against AlCl_3_ (200 μM) for 24 h. After incubation, CVDK-8 reagent was added to each well and incubated for 3 h. At the end of the incubation period, the absorbance of each sample was measured at 450 nm in a microplate reader (Synergy-HT; BioTek Winooski, VT, United States). As a positive control, cells were treated with 0.1% (*w/v*) Triton X-100.

#### LDH assay

Following the provider’s instructions, the LDH assay was performed using the CytoSelect™ LDH Cytotoxicity Assay Kit (Cell BioLabs, San Diego, CA, United States). Briefly, the cells were treated as mentioned above, and at the end of the culture period, 90 μL of supernatant was transferred to a new plate, and 10 μL of the reaction mixture was added to each well. The reaction was incubated for 30 min at room temperature in the dark. Eventually, the optical density was measured at a wavelength of 450 nm in a microplate reader (Synergy-HT; BioTek Winooski, VT, United States). As a positive control, cells were treated with 0.1% (*w/v*) Triton X-100 (24).

### Animals and *in vivo* experimental design

Adult male Wistar rats weighing 230 ± 20 g were procured from ATADEM, Ataturk University (Turkey). In the Experimental Animals Housing Unit facility in Atatürk University’s Faculty of Pharmacy, animals were maintained at room temperature (25°C) with a 12-h light/dark cycle. Rats were given a regular pellet diet and had unlimited access to food and water *ad libitum*. Before starting the medication therapy, the rats were allowed to acclimatize for a week.

The animal ethics committee of Ataturk University authorized the experimental protocol for the care of experimental animals (approval number 77040475-000-E.1800140631-1851, date of approval 26 April 2018). Animal handling and all procedures were performed in accordance with and strictly adhered to the “Guide for the Care and Use of Laboratory Animals” 8th edition.

Chronic administration of AlCl_3_ at various levels in mice has been utilized in various investigations to mimic the physiology of Alzheimer’s disease (25). In our study, AlCl_3_ was used at a dose of 5 mg/kg/i.p. for four weeks. This dosing regimen of AlCl_3_ was selected based on previous reports because of the high rate of induction and low mortality (26, 27).

A total of 39 rats were randomly divided into six groups:

**(1)**. **The control group (CG, *n* = 5)** received saline (1 mL/kg/day, i.p.) for four weeks.

**(2). The AD model group (*n* = 10)** was injected daily with AlCl_3_ (5 mg/kg/day, i.p.) for four weeks.

**(3). DCS group (*n* = 6):** Rats in this group received DCS (3.6 mg/kg/day i.p.) for four weeks.

**(4). LS group (*n* = 6):** Rats in this group received LS (3.6 mg/kg/day i.p.) for four weeks.

**(5). AlCl_3_ + DCS group (*n* = 6):** Rats in this group were induced with AlCl_3_ and subsequently received DCS (3.6 mg/kg/day i.p.) for four weeks.

**(6). AlCl_3_ + LS group (*n* = 6):** Rats in this group were induced with AlCl_3_ and subsequently received LS (3.6 mg/kg/day i.p.) for four weeks.

Four days before the study’s end date, rats were trained in the Morris water maze. On the last day of the study, animals received the last treatment dose, and the passive avoidance test was later performed. After 24 h, all animals were anesthetized with isoflurane and sacrificed. The blood samples were collected in both EDTA anticoagulant tubes and no anticoagulant tubes. The plasma was separated by centrifugation at 3000 rpm for 10 min at 4°C. The serum was separated from the blood. Whole blood samples were used for the hematological test, while plasma and serum samples were used for the biochemical analysis.

The brains were taken immediately, frozen in liquid nitrogen, and stored at –80°C. The brains were stored in neutral buffered formalin (pH-7.4) for histological investigations.

### Neurobehavioral studies

#### Morris water maze

Morris water maze procedures were used to test rats’ spatial memory and learning (28, 29). In this study, a circular swimming pool with a diameter of 150 cm and a height of 40 cm was divided into four quadrants (NW, NE, SE, and SW), with an escape platform located in the NW quadrant that remained 2 cm below the water level during the acquisition trials. External cues were set all over the pool and stayed the same throughout the trial. During the training days, the rats were taught to find this concealed platform by performing four acquisition trials each day for four days in a row (up to 90 seconds). The time needed for each rat to reach the platform was graphically recorded as the escape latency. Successful rats were permitted to stay on the platform for 10 s before being removed; however, if the rat did not find the platform within 60 s, it was gently directed to it and allowed to stay for another 15 s. The animals underwent four acquisition trials per day for four days in a row. The animal was placed in each quadrant during each experiment to remove quadrant effects. The trial time was reported as 2 min in the trials when the rats failed to reach the platform. On the fifth day, each rat was given a 90-s probe experiment in which the platform was withdrawn from the pool. The amount of time spent swimming in the target quadrant (within 90 s of the probe test time) was tracked.

#### Passive avoidance task

The passive avoidance task (PAT) is a widely used method for assessing the preservation of avoidance memory in mice. As reported in a prior study, a step-by-step PAT was carried out (30). The device had two bright and dark chambers, divided by an automatic door. The animal was placed in the light chamber for the acquisition session. The door was lifted after 30 s of acclimatization, and when the animal entered the dark compartment, a modest electric shock of 0.5 mA was provided for 3 s. After a 24 h acquisition trial, a retention trial was conducted using the same approach as the acquisition trial but without the use of electric shock. Each mouse’s transfer latency time (sec) was collected in both the acquisition and retention trials. The test was stopped if the rat did not enter the dark room during the 5 min test period, and the step-through latency was recorded as 300 s (31).

#### Histopathological examination

Brain tissues of treated and control rats were fixed in 10% buffered formalin solution in labeled bottles. Tissues were stained with hematoxylin-eosin (H-E) and examined under a microscopic imaging system (Leica Microsystems GmbH, Wetzlar, Germany).

#### Biochemical and hematological assays

An automated analyzer (Archem, BM240, Istanbul, Turkey) was used to assay for biochemical and hematological parameters.

#### Total oxidative stress and total antioxidant capacity analysis

Total antioxidant capacity (TAC) assays and total oxidant status (TOS) assays were conducted to measure antioxidative/oxidative capacity in the brain using commercially available TAC and TOS assay kits (Rel Assay Diagnostics^®^, Gaziantep, Turkey). Ascorbic acid (10 μM) and hydrogen peroxide (25 μM) from Sigma–Aldrich were used as positive control treatments to determine TAC and TOS levels, respectively (32).

#### Real-time PCR analysis

RNA isolation was performed by homogenizing brain tissues with a Pure Link™ RNA Mini Kit (Invitrogen™, Carlsbad, CA, United States) following the provider’s manual. Then, cDNA synthesis was conducted using 10 μL of RNA with a High-Capacity cDNA Reverse Transcription Kit (Applied Biosystems™, United States) following the provider’s manual. qPCR was carried out using Sybr Green Master Mix (Applied Biosystems™, United States) on a Real-Time PCR Detection System (Qiagen Rotor-Gene Q). The qPCR program was 50°C for 2 min, 95°C for 10 min x 40 cycles, 95°C for 15 s, and 60°C for 1 min (33). mRNA expression levels were normalized to ACTB mRNA expression levels. A list of the primers used is given in Supplementary Table 1.

#### Statistical analyses

Statistical analysis was conducted using the SPSS^®^ 21.0 program. The results are given as the mean ± standard deviation. Duncan’s test was used as a *post hoc* test followed by a one-way analysis of variance (ANOVA). *P* < 0.05 was set as the minimal level of significance.

## Results

### D-cycloserine and L-serine protect differentiated SH-SY5Y cells from damage induced by AlCl_3_

**FIGURE 1:**
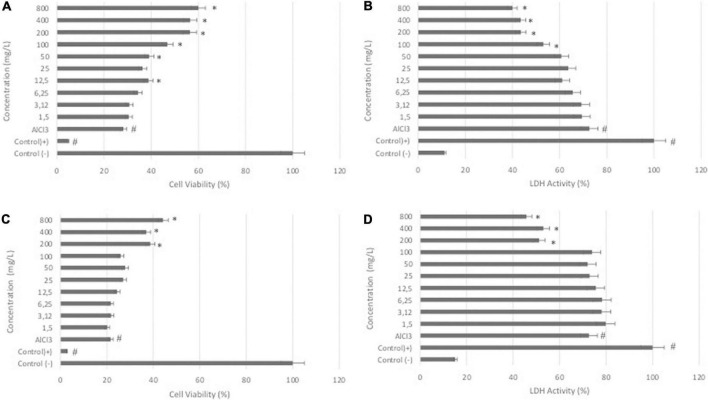
Effects of DCS and LS against AlCl_3_-induced neurotoxicity in differentiated SHSY-5Y cells. **(A)** Viability of differentiated SHSY-5Y cells after 24 h of DCS (0-800 μg/ml) and AlCl_3_ treatment. **(B)** LDH activity of cells after 24 h of DCS (0-800 μg/ml) and AlCl_3_ treatment. **(C)** Viability of differentiated SHSY-5Y cells after 24 h of LS (0-800 μg/ml) and AlCl_3_ treatment. **(D)** LDH activity of cells after 24 h of LS (0-800 μg/ml) and AlCl_3_ treatment. All values are expressed as the mean ± standard deviation. Significance difference between groups indicated by: # between control and AlCl_3_, * between AlCl_3_ and treatment groups.

The results of the WST-8 assay showed that the treatment of differentiated SH-SY5Y cells with DSC or LS at different concentrations (0-800 μg/ml) for 24 h had no significant effect on cell viability (data not shown). In differentiated SH-SY5Y cells, treatment with AlCl_3_ at a concentration of 200 μg/ml dramatically reduced the cell viability rate (*P* < 0.05). Cotreatment with DCS or LS, on the other hand, resulted in a substantial increase in percent cell viability, showing that DCS and LS have a neuroprotective impact (Figures 1A,C) (*P* < 0.05). The LDH assay confirmed the WST-8 results that demonstrated that cell membrane integrity was affected by ALCl_3_ in a similar pattern. LDH activity was increased in the supernatant of AlCl_3_-treated cells compared with untreated cells. The protective effect of DCS and LS against AlCl_3_-induced toxic effects was also confirmed in the LDH assay (Figures 1B,D) (*P* < 0.05).

### D-cycloserine and L-serine attenuated AlCl_3_-induced learning and memory deficits

**FIGURE 2:**
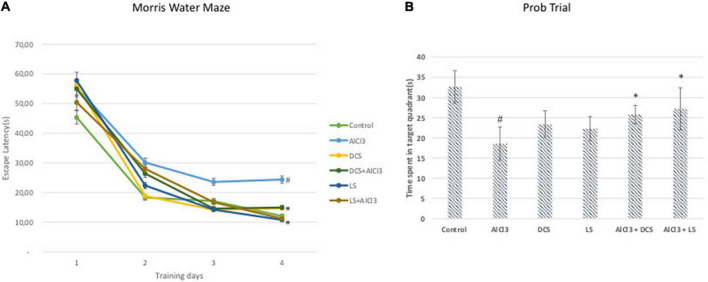
**(A)** Escape latency in the MWM test of each training day. **(B)** Mean time spent in the target quadrant in the MWM test. The behavioral analysis **(A,B)** was compared to the control group. Significance difference between groups indicated by: # between control and AlCL_3_, * between AlCl_3_ and treatment groups.

The AlCl3-treated group displayed a significantly (*P* < 0.05) longer escape latency than the untreated control group in the Morris water maze test. In contrast, when compared to the disease-control group, DCS or LS treatment significantly (*P* < 0.05) reduced the rise in escape latency brought on by aluminum chloride treatment (Figure 2).

**FIGURE 3:**
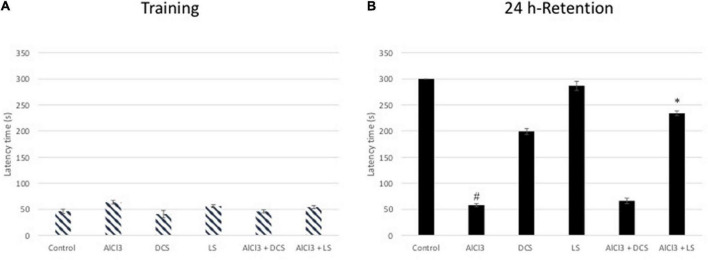
Performance in the passive avoidance test. The training latencies **(A)** and the retention latencies **(B)** to enter the dark chamber during sessions are shown. All values are expressed as the mean ± standard deviation. Significance difference between groups indicated by: # between control and AlCl_3_, * between AlCl_3_ and treatment groups.

The transfer delay for each mouse was assessed during the passive avoidance test for both the acquisition and retention phases. In the acquisition test, there was no noticeable difference in the transfer latency between any of the experimental groups, according to one-way ANOVA statistical analysis. However, in the retention test, the aluminum chloride-treated group showed a highly significant decline compared to the healthy control group. The DCS treatment demonstrated a considerable improvement in retention latency compared to the disease control group. Compared to DCS, treatment with LS had more notable results (Figure 3).

### D-cycloserine and L-serine attenuated the generation of neurofibrillary tangles in the AlCl_3_-induced AD rat brain

**FIGURE 4:**
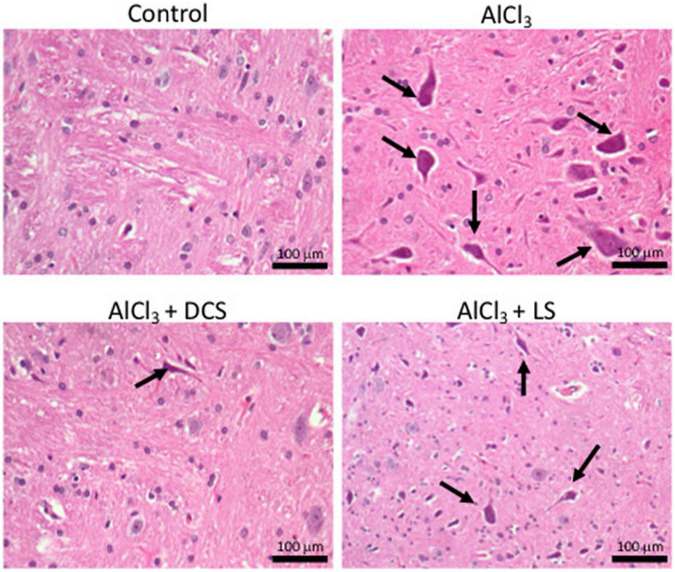
Representative histopathology images in the rat brain (*n* = 3) (hematoxylin-eosin, original magnification 200×) Arrows indicate neurofibrillary tangles.

Figure 4 displays the results of the histopathological tests performed on the brain tissues of the rats in the control and experimental groups using hematoxylin and eosin staining. The figure depicts the accumulation of neurofibrillary tangles in the brains of rats exposed to AlCl_3_ (indicated by arrows). Recovery in the pathogenic alterations in the brain tissue was seen after treatment with DCS or LS.

### D-cycloserine and L-serine attenuated aspartate aminotransferase, alanine aminotransferase, and creatine kinase levels

The toxicology results for the hematological parameters are shown in Supplementary Table 2. According to the findings, there was a statistically significant increase in aspartate aminotransferase (AST) and alanine aminotransferase (ALT) and a significant decrease in creatine kinase (CK) and uric acid values (*P* < 0.05) in AlCl_3_-treated animals. Additionally, cotreatment with DCS or LS led to amelioration of these negative changes caused by AlCl_3_ in rats.

### D-cycloserine reduces AlCl_3_-Induced inflammation

As depicted in Supplementary Table 3, AlCl_3_ led to a significant increase in the level of neutrophils and a decrease in basophils compared to the control group (*P* < 0.05). Most importantly, DCS treatment opposed the effect of ALCl_3_ and lowered neutrophil levels compared to the AlCl_3_-treated group.

**FIGURE 5:**
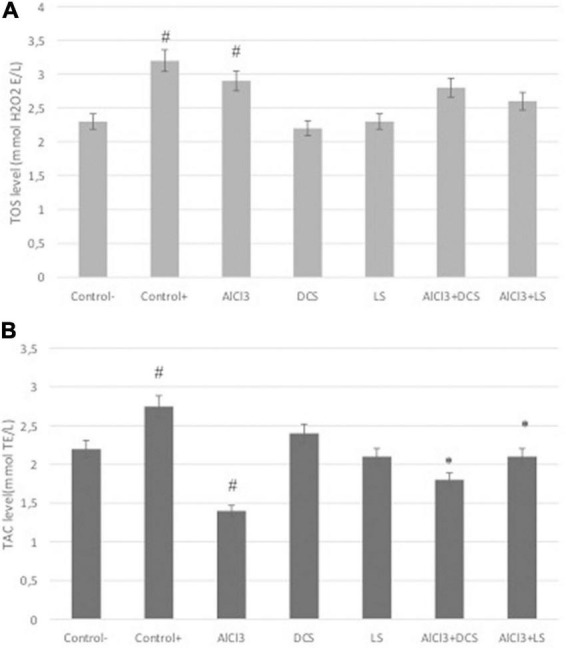
**(A)** Total antioxidant capacity (TAC) and **(B)** total oxidative stress (TOS) levels in all groups. The data are expressed as the mean ± standard deviation. Significance difference between groups indicated by: # between control and AlCl_3_, * between AlCl_3_ and treatment groups.

Additionally, the mRNA expression of TNF was markedly increased by AlCl3 incubation relative to the control group (Figures 5C,D). Compared with the AlCl_3_-treated group, DCS and LS treatment significantly decreased the mRNA expression of TNF-a.

### D-cycloserine and L-serine attenuated the decreased levels of TAC

Total antioxidant capacity and total oxidative status were measured in brain tissue samples from the rat groups, and the results are shown in Figure 5. The results revealed that both DCS and LS support TAC levels. As a positive control, DCS, LS and ascorbic acid (10 μM) increased TAC levels by approximately 2.37-, 2.16- and 2.75-fold, respectively. At the TOS level, hydrogen peroxide (25 μM), used as a positive control, caused an approximately 3.12-fold increase, while AlCl_3_ caused a 2.8-fold increase. However, the DCS- and LS-treated groups did not exhibit changes in TOS levels compared to the untreated group. The results showed that AlCl_3_ exposure caused a significant (*P* < 0.05) decrease in TAC levels and an increase in TOS levels. In addition, it has been shown that the negative change in TAC levels caused by ALCl_3_ is alleviated by DCS and LS applications. LS was found to be more effective at alleviating oxidative stress induced by AlCl_3_ than DCS.

### D-cycloserine and L-serine decreased amyloid-beta production in the AD rat model

**FIGURE 6:**
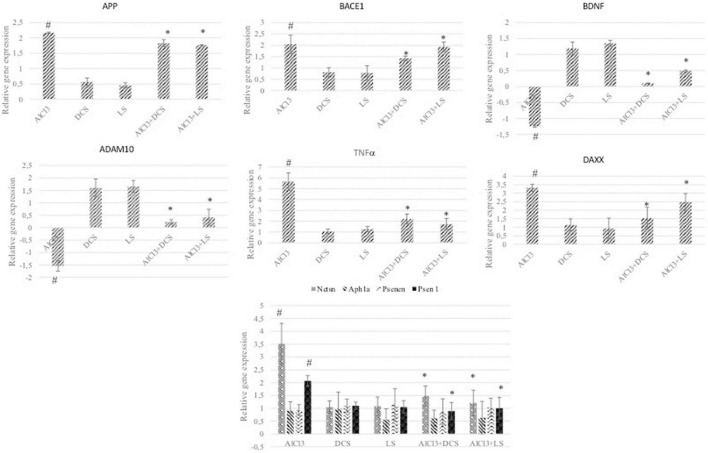
The graphs represent the relative expression levels of different genes related to Alzheimer’s disease. The analyzed genes were APP, BACE1, BDNF, ADAM10, TNFα, DAXX, NCTSN, APH1A, PSENEN, and PSEN1. The data are expressed as the mean ± standard deviation. Significance difference between groups indicated by: # between control and AlCl_3_, * between AlCl_3_ and treatment groups.

To further analyze the protective roles of DCS and LS against AlCl3-induced neurotoxicity, the levels of Aβ metabolism-related genes, such as APP, BACE 1, NCTSN, PSEN1, PS, ADAM10, and APH1A, were evaluated by RT–PCR. RT–PCR analysis revealed that exposure to AlCl3 significantly increased the mRNA expression of APP, BACE 1, NCTSN, and PSEN1 and inhibited the mRNA expression of ADAM10 relative to the control group (*P* < 0.05). Additionally, treatment with DCS or LS showed a significant (*P* < 0.05) beneficial effect in counteracting the effect of AlCl3 in the treated brain tissues (Figure 6).

## Discussion

The current findings show that DCS and LS protect rats from AlCl_3_-induced Alzheimer-like pathology. One of the most common clinical signs of Alzheimer’s disease is cognitive loss, and aluminum causes most of the disease’s symptoms (34–36). Aluminum causes behavioral, physiological, and neurochemical changes, which eventually contribute to cognitive impairment, as seen in Alzheimer’s disease (37–39). We found that DCS and LS reduced behavioral, biochemical, and neurochemical abnormalities in AlCl_3_-treated rats, indicating that they might have a neuroprotective function in Alzheimer’s disease.

Chronic exposure to AlCl_3_ resulted in a significant reduction in memory retention and spontaneous memory impairment as measured by passive avoidance and the Morris water maze (MWM) test, respectively. The results were in agreement with previous studies (26, 27). DCS or LS coadministration reduced the cognitive impairment caused by chronic AlCl_3_ exposure, demonstrating that DCS and LS are powerful neurostimulators and memory enhancers. In the case of aluminum and oxidative stress, it has been established that oxidative damage is responsible for the etiology and cognitive dysfunctions in Alzheimer’s disease (40). The total oxidant status (TOS) is usually used to estimate the overall oxidation state of the sample. Similarly, the total antioxidant status (TAS) is used to measure the overall antioxidant status of the sample. Because the measurement of different antioxidant molecules separately is not practical and their antioxidant effects are additive, we preferably measured the total antioxidant capacity and the total oxidant status (41). According to this study, chronic aluminum exposure reduces TAC levels in rat brain homogenates. It has been previously reported that aluminum treatment causes neurochemical changes in various brain areas as well as changes in the brain’s oxidative state, which is in line with our findings (42). Thus, prolonged aluminum exposure disrupts the balance between antioxidants and oxidative processes, a condition that is most likely responsible, at least in part, for the observed memory impairment in rats.

We then examined blood parameters to determine whether there were any changes in the hematological and biochemical systems. The value of CK was found to be significantly lower in AlCl_3_-treated rats. Creatine kinase, which is vulnerable to oxidative damage and is significantly diminished in AD brains (43), causes a shift in glutamate concentrations and cellular toxicity. In contrast, oxidative damage to creatine kinase may affect energy equilibrium in the brain. The findings also revealed that DCS or LS therapy significantly increased CK activity, which was inhibited by AlCl_3_ treatment. The reason could be the antioxidant characteristics of L-serine, as previously documented (44). L-serine is likely implicated in the cellular antioxidant defense system because its downstream metabolites glycine and cysteine are precursor amino acids necessary for the formation of the antioxidant glutathione (GSH), which shields cells from oxidative damage (45, 46) L-serine treatment has been found to have promising therapeutic benefits on brain damage and ischemic stroke in preclinical investigations (47, 48).

Alzheimer’s disease is characterized pathologically by inflammation, and immune cells have been implicated in the pathophysiology of the disease. Neutrophils, well-known players of the immune system, perform a variety of tasks, such as producing reactive oxygen species (ROS), phagocytosis, degranulation, and releasing neutrophilic extracellular traps (NETs) (49–51). Neutrophils have a role in the pathophysiologic processes of AD, and the disease process itself may cause an increase in neutrophil count, according to a number of suggested pathways. Tumor necrosis factor-alpha (TNF-α) is a cytokine that is significantly increased in Alzheimer’s disease (AD) and is closely associated with the development of neuropsychiatric symptoms (52). Additionally, it is known that TNF promotes neutrophil survival by releasing IL-9 through an NF-ß-dependent mechanism (53). This might be the reason why people with AD have higher neutrophil levels. Basophils express the high-affinity IgE receptor FcRI and contain histamine (54–56). Additionally, it has been shown that AD patients have higher quantities of histamine, a neurotransmitter with anti-inflammatory properties, in their brains and serum. However, it is still unclear how AD affects the quantity and functionality of basophils (57).

In previous investigations, NMDA neurotransmission enhancers have been shown to ameliorate behavior and memory symptoms (58, 59). However, there is disagreement regarding whether D-cycloserine (a partial agonist of the NMDAR-glycine site) might enhance cognitive performance in dementia patients (21, 22, 60, 61). Clinical investigations have found that using significant amounts of D-serine and D-cycloserine as adjuvant therapy in schizophrenia patients can help with positive, negative, and cognitive symptoms (62, 63). Several studies have examined the link between AD and D-serine levels in serum or CSF. The outcomes were contentious. According to a previous study with a smaller sample size, d-serine serum levels in Alzheimer’s patients were somewhat lower than those in normal controls (64). In more recent research, D-serine levels were shown to be increased in postmortem AD brains and CSF of probable AD patients, although the findings were not validated in other studies (65, 66). In contrast to previous studies that enrolled medicated AD patients, a newly published cohort study with a larger sample size that enrolled the entire clinical spectrum of drug-free AD patients revealed indistinguishable CSF and serum D-serine levels and D-serine/total serine ratios compared to controls (67).

The liver enzymes in the animal model of Alzheimer’s disease showed significant elevations. The aminotransferases AST, OT1, and ALT (PT1), released into the bloodstream when the liver is damaged, are the most sensitive and widely used diagnostic liver enzymes. As a result, an increase in these enzymes indicates widespread hepatocyte death (hepatic necrosis), which is seen in many inflammatory illnesses (68, 69). In 90% of ischemia or toxic liver injury cases, high levels of aminotransferases have been observed (70). According to one study, blood AST levels increased 6–7 times in 98% of alcoholic hepatitis patients compared to normal values (71).

The amyloid cascade hypothesis establishes that aberrant Aβ aggregation in the brain is the primary cause of Alzheimer’s disease, which arises when Aβ production and clearance are out of balance. In the amyloidosis route, Aβ1-42 is generated when APP is sequentially cleaved by β and γ secretases; however, in the non-amyloid pathway, APP is cleaved by α and γ secretases, which avoids Aβ production and seems to be a protective process (72). Previous studies have found that prolonged exposure to AlCl_3_ increased the expression of APP, Aβ1-42, β and γ secretases, which accelerated Aβ formation and decreased its degradation (35, 73). Aluminum has been shown to accelerate Aβ production and aggregation, cause structural changes in Aβ, and promote the creation of Aβ oligomers (74–76). Exley (77) found that Al raises the Aβ burden in experimental animals by affecting Aβ anabolism or catabolism. The present study indicated that genes associated with Aβ metabolism, such as ADAM10, BACE1, PS1, and NCT, are involved in aluminum-induced neurotoxicity. It can be said that aluminum increases β-secretase and γ-secretase activities. Moreover, DCS or LS attenuated Al-induced Aβ toxicity by lowering the expression of APP, β- and γ-secretases.

The DAXX protein is associated with the FAS protein, which belongs to the tumor necrosis factor receptor superfamily and contains the death domain critical for apoptotic signaling. Overexpression of DAXX induces apoptosis, and its expression has been reported to be increased in the AD brain in previous studies (78). As shown in Figure 6, DAXX expression was increased in the AlCl_3_-treated group compared to the control. In addition, DAXX expression decreased significantly due to DCS and LS applications. This finding shows that aluminum causes AD-related pathogenesis by causing apoptosis and that DCS and LS are protective against AlCl_3_-induced apoptosis.

The failure of therapeutic tactics directed at a single component of Alzheimer’s disease is apparent, and there is mounting evidence that the development of successful therapies for the disorder must consider this. In this context, the use of preventative medications, such as neuro-nutrients that can slow the progression of Alzheimer’s disease is becoming more popular (79). This research supports this viewpoint, demonstrating that LS can have various therapeutic benefits and potentially contribute to current and future anti-AD treatments.

## Data availability statement

The datasets presented in this study can be found in online repositories. The names of the repository/repositories and accession number(s) can be found in the article/Supplementary material.

## Ethics statement

The animal study was reviewed and approved by the Animal Ethics Committee of Ataturk University.

## Author contributions

ÖT, UO, and OC carried out the experiment. ÖT and CB wrote the manuscript with support from AH, AM, and HT conceived the original idea and supervised the project. All authors contributed to the article and approved the submitted version.
