# Supplementary material for: Assessment of the neuroprotective potential of d-cycloserine and l-serine in aluminum chloride-induced experimental models of Alzheimer’s disease: In vivo and in vitro studies
Source: Front Nutr. 2022 Sep 8;9:981889. doi: 10.3389/fnut.2022.981889 (PMC9493202; doi:10.3389/fnut.2022.981889)
Supplement: Supplementary file 1 [file Data_Sheet_1.docx]

**S1**. Primer list used for RT-PCR Analysis

| Gene | Forward | Reverse |
| --- | --- | --- |
| Psen1 | TGCTCTTTGTTCCTGTGACC | TCGGTGTCTTCTGTGAATGG |
| Mapt (Tau) | CAGTCGAAGATTGGCTCCTT | GTGGAGATGTGTCCCCAGAC |
| App | GGTGGAGCGGACACAGACTA | ACATCCTCGTCATCCTCAGC |
| Adam10 | TGCCTCCCAAAGTCTCTCAT | AGACGTTGCTCTCGCATACA |
| Bace1 | ACTGTGCGTGCCAACATT | GCTTCACCAGGGAGTCAAAA |
| Psenen | TGCCTTTTCTTTGGTTGGTC | TGATCCAGGTGGTGAGAACA |
| Egfr | TCATCAGGGGAAATGCTCTC | GAGGATGGGGTTGTTGCTAA |
| Apoe | ACCGCTTCTGGGATTACCTG | GTGTCTCCTCCGCCACTG |
| Bdnf | ACAGTATTAGCGAGTGGGTCAC | GAACATACGATTGGGTAGTTCG |
| Daxx | \|  \| GGGATGTGGGTGTCAGGTTA \| \| --- \| --- \| | GGTGAGTGCGGGGTCAAC |
| Vegfa | CCCTGGTGGACATCTTCC | TGGCTTTGGTGAGGTTTGAT |
| APH1A | TCCTGCTCCATACCTTTTGG | CTGTGACGAATGCCCAGAG |
| Tnf | TGATCCGAGATGTGGAACTG | ATGAGAGGGAGCCCATTTG |
| NCSTN | ATTGTGTGGGGGAAACTCAG | CCGTCAATACCCATTTCAGG |
| Actb | CCAACCGTGAAAAGATGACC | TACGACCAGAGGCATACAGG |

**S2.** Haematology parameters of rat blood samples.

|  | **Control** | **AlCl_3_** | **DCS** | **LS** | **AlCl_3_ + DCS** | **AlCl_3_ + LS** |
| --- | --- | --- | --- | --- | --- | --- |
| WBC (10^9/L) | 4,34 | 4,74 | 4,21 | 5,78 | 5,10 | 4,77 |
| LYMPH(%) | 80,30 | 81,00 | 82,50 | 81,50 | 80,50 | 82,50 |
| NEUT(%) | 12,60 | 123,00 | 11,14 | 9,35 | 74,25 | 117,00 |
| MONO(%) | 1,50 | 1,40 | 1,00 | 1,21 | 1,20 | 1,60 |
| EO(%) | 5,40 | 6,30 | 3,50 | 4,22 | 4,80 | 4,25 |
| BASO(%) | 3,20 | 0,54 | 2,25 | 2,10 | 1,20 | 0,47 |
| RBC(10^12/L) | 5,41 | 6,91 | 5,95 | 6,07 | 6,41 | 4,37 |
| HGB(g/dL) | 9,60 | 11,40 | 10,50 | 11,50 | 11,50 | 8,20 |
| HCT(%) | 32,40 | 30,39 | 33,70 | 31,57 | 39,70 | 32,25 |
| MCH(pg) | 17,70 | 16,50 | 17,60 | 18,00 | 17,20 | 18,80 |
| MCV(fL) | 59,90 | 57,60 | 56,60 | 56,60 | 59,10 | 58,80 |
| MCHC(g/L) | 29,60 | 28,60 | 31,20 | 31,70 | 29,20 | 31,90 |
| RDW-SD | 26,10 | 21,10 | 24,90 | 23,50 | 25,20 | 23,10 |
| RDW-CV(%) | 15,90 | 18,90 | 13,00 | 14,60 | 16,50 | 16,80 |
| MPV(fL) | 8,00 | 8,00 | 7,70 | 8,40 | 8,10 | 8,70 |
| PLT(10^9/L) | 767,00 | 506,00 | 565,00 | 712,00 | 726,00 | 734,00 |
| PCT(%) | 0,60 | 0,40 | 0,40 | 0,60 | 0,70 | 0,50 |
| PDW(fL) | 7,80 | 8,70 | 7,40 | 8,20 | 9,20 | 9,40 |
| NRBC (%) | 1,50 | 1,00 | 1,01 | 1,70 | 1,20 | 1,60 |

**S3.** Clinical biochemistry parameters of rat blood samples.

|  | **Control** | **AlCl_3_** | **DCS** | **LS** | **AlCl_3_ + DCS** | **AlCl_3_ + LS** |
| --- | --- | --- | --- | --- | --- | --- |
| CK (U/L) | 682,00 | 277,00 | 570,00 | 580,00 | 443,00 | 351,00 |
| AST(U/L) | 139,00 | 168,00 | 132,00 | 160,00 | 156,00 | 140,00 |
| ALT(U/L) | 48,00 | 62,00 | 51,00 | 54,00 | 54,00 | 48,00 |
| LDH (U/L) | 469,00 | 598,00 | 419,00 | 463,00 | 412,00 | 400,00 |
| Triglyceride(mg/dL) | 61,00 | 60,00 | 66,00 | 62,00 | 64,00 | 70,00 |
| Total Cholesterol(mg/dL) | 68,00 | 64,00 | 77,00 | 80,00 | 71,00 | 62,00 |
| Ca(mg/dL) | 11,20 | 9,80 | 8,90 | 10,3 | 10.70 | 8,10 |
| P (mg/dL) | 8,00 | 7,90 | 6,70 | 7,80 | 8,90 | 7,80 |
| Mg(mg/dL) | 3,25 | 2,26 | 2,81 | 2,45 | 2,49 | 2,73 |
| Total Bilirubin(mg/dL) | 0,31 | 0,01 | 0.54 | 0,45 | 0.05 | 0,06 |
| Crea. (mg/dL) | 0,50 | 0,30 | 0,20 | 0,30 | 0,30 | 0,50 |
| Na (mmol/L) | 135,00 | 125,00 | 125,00 | 126,00 | 129,00 | 130,00 |
| K(mmol/L) | 12,45 | 18,48 | 12,36 | 14,25 | 13,82 | 14,84 |
| Uric Acid (mg/dL) | 7,50 | 4,50 | 6.30 | 6,70 | 5,00 | 6,10 |
| BUN(mg/dL) | 24,77 | 21,03 | 24,49 | 21,50 | 21,16 | 23,36 |
